# Irigenin Modulates BL‐Induced Pyroptosis in Retinal Pigment Epithelial Cells Through p38 MAPK and NFκB Pathways

**DOI:** 10.1002/jbt.70723

**Published:** 2026-02-05

**Authors:** I‐Li Su, Kun‐Lin Yeh, Chien‐Ying Lee, Sheng‐Chien Lin, Chen‐Yu Chiang, Chun‐Jung Chen, Wen‐Ying Chen, Ching‐Chi Tseng, Yin‐Che Lu, Yu‐Hsiang Kuan

**Affiliations:** ^1^ Department of Cardiovascular Surgery Antai Medical Care Corporation Antai Tian‐Sheng Memorial Hospital Pingtung Taiwan; ^2^ Department of Veterinary Medicine, College of Veterinary Medicine National Chung Hsing University Taichung Taiwan; ^3^ Department of Pharmacology, School of Medicine Chung Shan Medical University Taichung Taiwan; ^4^ Department of Pharmacy Chung Shan Medical University Hospital Taichung Taiwan; ^5^ Department of Education and Research Taichung Veterans General Hospital Taichung Taiwan; ^6^ Department of Dermatology The Wilshire Lab and Aesthetic Clinic Shenzhen China; ^7^ Department of Dermatology Shiso Municipal Hospital Hyogo Japan; ^8^ Division of Hematology‐Oncology Ditmanson Medical Foundation Chia‐Yi Christian Hospital Chiayi Taiwan

**Keywords:** A2E, BL, inflammation, Irigenin, pyroptosis, retinal pigment epithelial cell

## Abstract

Age‐related macular degeneration (AMD), a primary cause of vision loss among older adults, is strongly associated with inflammatory processes. The current study aimed to elucidate the protective effects of irigenin, an isoflavonoid recognized for its anti‐inflammatory, antioxidative, antiapoptotic, and anticancer activities, against blue light (BL)‐induced damage in N‐retinyl‐N‐retinylidene ethanolamine (A2E)‐laden human adult retinal pigment epithelial (A2E‐laden ARPE‐19) cells. Pretreatment with irigenin markedly mitigated BL‐induced cytotoxicity and preserved epithelial barrier function in a concentration‐dependent manner. Moreover, irigenin significantly inhibited the expression of proinflammatory cytokines and activation of the nod‐like receptor pyrin domain‐containing 3 (NLRP3) inflammasome, as evidenced by decreased expression of NLRP3, ASC, and both full‐length and cleaved forms of gasdermin D (GSDMD), along with reduced caspase‐1 activity. Further mechanistic analyses indicated that irigenin effectively suppressed the activation of the nuclear factor kappa B (NFκB) signaling pathway, as evidenced by phosphorylation of NFκB and inhibitor of NFκB (IκB)α, and both activation and translocation of NFκB, along with reduced phosphorylation of p38 mitogen‐activated protein kinase (MAPK). These findings underscore the potential of irigenin to ameliorate BL‐induced retinal pigment epithelial cell damage via modulation of inflammation and pyroptosis pathways, suggesting its therapeutic value for preventing AMD.

## Graphical abstract

Irigenin alleviates blue light‐induced RPE cell damage by inhibiting NLRP3 inflammasome and p38/NF‐κB signalling, showing potential for AMD prevention.

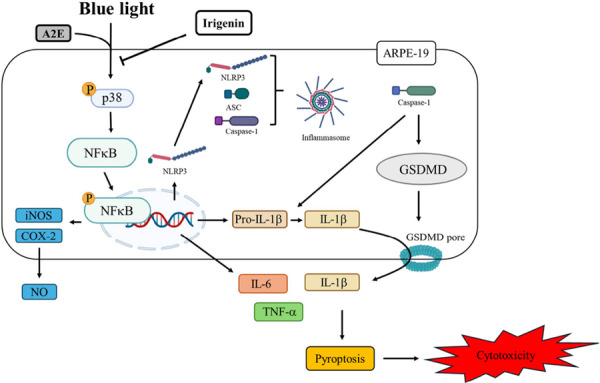

## 1 Introduction

AMD, a leading cause of blindness among older individuals, is marked by progressive and irreversible vision loss [1]. The pathogenesis of AMD involves the complex interplay of aging, environmental factors, and genetic predispositions, with chronic inflammation and oxidative stress being key drivers of its progression [2]. In addition to classical apoptotic and necrotic pathways, recent evidence suggests that pyroptosis, an inflammation‐driven form of programmed cell death, is critical to AMD progression [3]. Retinal photoreceptors are essential for initiating the visual process but highly susceptible to BL damage, which is exacerbated by A2E, a phototoxic byproduct of retinal turnover that accumulates in retinal pigment epithelium (RPE) cells [4]. BL irradiation triggers the formation of reactive oxygen species (ROS) via A2E photoactivation and other cytotoxic compounds, increasing oxidative stress and triggering inflammasome activation [5]. This initiates an inflammatory cascade leading to pyroptosis, a lytic cell death mechanism regulated by innate immune perturbations and characterized by the release of proinflammatory mediators like IL‐1β [6]. Although pyroptosis initially serves as a defense mechanism against pathogens, its chronic activation promotes sustained inflammation, which is increasingly implicated in AMD [7]. Recent evidence suggests that pyroptosis heightens RPE cell susceptibility to photooxidative damage, fostering degeneration and underscoring its potential role as a significant driver in AMD pathogenesis [8].

Because of the multifactorial nature of AMD pathogenesis, various natural compounds are under investigation for their potential to mitigate inflammation, oxidative stress, and retinal degeneration [9]. These agents, particularly botanical isoflavones, have demonstrated protective effects through modulation of inflammatory pathways, reduction of oxidative damage, and attenuation of cell death processes [10]. Among these, irigenin, an isoflavone isolated from *Belamcanda chinensis* (L.) DC., stands out for its potent anti‐inflammatory and antioxidant properties, previously shown to confer benefits in models of cancer, diabetes‐related complications, inflammatory conditions, and cardiac injury [11]. Despite these insights, the potential of irigenin in retinal diseases such as AMD remains largely uncharted. This investigation utilized an in vitro model of dry AMD through the application of A2E‐laden ARPE‐19 cells. This model effectively simulates the oxidative and inflammatory stress experienced by RPE cells during the early stages of atrophic AMD [12, 13, 14]. However, it is essential to acknowledge that this model does not adequately replicate the pathological neovascularization associated with wet AMD, as it lacks both choroidal endothelial cells and the requisite angiogenic stimuli [15, 16, 17]. Therefore, the results and interpretations of this study are limited to the dry form of AMD and cannot be extrapolated to angiogenesis‐related mechanisms in wet AMD. This study was designed to explore the potential of irigenin in mitigating BL‐induced inflammation and pyroptosis.

## 2 Materials and Methods

### 2.1 Cell Culture

The human retinal pigment epithelial cell line ARPE‐19 was obtained from the Bioresource Collection and Research Center, Food Industry Research and Development Institute (Hsinchu, Taiwan) and cultured in Dulbecco's Modified Eagle Medium (HyClone Laboratories, Logan, UT, USA) with the addition of 10% fetal bovine serum (HyClone Laboratories, Logan, UT, USA) and antibiotics (HyClone Laboratories, Logan, UT, USA) [18].

### 2.2 Drug Treatment and BL Exposure

Cells were pretreated with 20 μM A2E for 24 h. After pretreatment, cells were incubated with irigenin (12.5, 25, or 50 μM) for 1 h, followed by exposure to BL irradiation (430 nm, 6000 lux) for 15 min. After light exposure, cells were returned to standard growth conditions and incubated for 24 h [19].

### 2.3 Lactate Dehydrogenase Assay

Cytotoxicity was determined by measuring lactate dehydrogenase (LDH) release using a commercially available assay kit (Thermo Fisher Scientific, Waltham, MA, USA), performed according to the provided protocol. After the treatment, cell culture supernatant was collected from each well and transferred to a new 96‐well plate. After adding the LDH detection solution, samples were incubated in darkness at room temperature for 30 min, and absorbance was subsequently measured at 490 and 680 nm with a microplate reader (BioTek, Winooski, VT, USA) [20].

### 2.4 Determination of Transepithelial Resistance

Cells were seeded onto transwell inserts with permeable membranes (0.4 µm pore size) and cultured until a confluent monolayer was established. Transepithelial electrical resistance (TEER) was measured using a volt‐ohmmeter from Kanto Chemical. Before each measurement, electrodes were equilibrated in PBS to ensure accuracy. During measurement, electrodes were placed in the apical and basal chambers of the Transwell system, and resistance values were recorded. Final TEER values were calculated as resistance per square centimeter (Ω·cm^2^) after adjusting for background membrane resistance, with values normalized to baseline to monitor changes in barrier integrity throughout treatment [21].

### 2.5 Griess Assay

After the treatment, the culture supernatants were collected and transferred into 96‐well plates, to which Griess reagent (composed of 1% sulfanilamide and 0.1% N‐(1‐naphthyl)ethylenediamine dihydrochloride dissolved in 2.5% phosphoric acid) was added. The mixtures were incubated for 10 min at room temperature in darkness, after which absorbance was recorded at 540 nm using a microplate reader. (BioTek, Winooski, VT, USA) [22].

### 2.6 Nuclear Factor Kappa Beta Activity Measurements

NFκB activation in A2E‐laden ARPE‐19 cells was evaluated using an NFκB transcription factor assay kit (Cayman, Ann Arbor, MI, USA). Nuclear extracts were prepared by incubating cells with permeabilisation buffer at 4°C for 10 min and 0.1% NP‐40 lysis buffer. After centrifugation, the nuclear fraction was isolated, resuspended in an extraction buffer, and kept on ice for 30 min. The extract was centrifuged, and the supernatant was collected for analysis. The nuclear extract was subsequently incubated with an oligonucleotide containing the NFκB binding site. NFκB activity was quantified using horseradish peroxidase–conjugated antibodies, and absorbance was measured at 450 nm (BioTek, Winooski, VT, USA).

### 2.7 Caspase‐1 Activity Measurements

Caspase‐1 activity was measured using a fluorometric assay per the manufacturer's instructions (Abcam, Cambridge, UK). After the treatment, cells were lysed in an ice‐cold buffer at 4°C for 1 h. Ac‐Tyr‐Val‐Ala‐Asp 7‐amino‐4‐trifluoromethylcoumarin (YVAD‐AFC) was used as a substrate to detect caspase‐1 activity. The fluorescence signals were recorded using a microplate reader (excitation: 400 nm, emission: 505 nm; BioTek Instruments, Winooski, VT, USA).

### 2.8 Enzyme‐Linked Immunosorbent Assay

IL‐1β, IL‐6, and tumor necrosis factor (TNF)‐α levels were measured using commercial enzyme‐linked immunosorbent assay (ELISA) kits according to the manufacturer's protocols (Thermofisher, Waltham, MA, USA). After treatment, supernatants were collected and loaded onto anti‐cytokine antibody‐coated plates for 16 h at 4°C. The plates were washed three times with phosphate‐buffered saline containing 0.05% Tween‐20 and then incubated with biotin‐conjugated anti‐cytokine antibodies for 1 h. Following additional washes, streptavidin‐horseradish peroxidase and 3,3',5,5'‐tetramethylbenzidine substrate solutions were added. The reaction was stopped with sulfuric acid, and absorbance was measured at 450 nm using a microplate reader (BioTek, Winooski, VT, USA) [23].

### 2.9 Western Blot Analysis

The cells were lysed in a radioimmunoprecipitation assay buffer with protease and phosphatase inhibitors to prevent protein degradation. The lysates were clarified through centrifugation at 12,000 rpm for 15 min at 4°C, and the resulting protein concentrations were quantified using a Bradford assay. Equal protein amounts were loaded onto sodium dodecyl sulphate–polyacrylamide gels for electrophoresis and transferred to polyvinylidene difluoride membranes. Membranes were blocked with 5% nonfat milk for 1 h at room temperature, followed by overnight incubation with primary antibodies at 4°C. horseradish peroxidase ‐conjugated secondary antibodies were applied for 1 h at room temperature. Primary antibodies, including iNOS, COX‐2, β‐actin, NLRP3, ASC, GSDMDC1, p‐NFκB, NFκB, p‐IκB, p‐p38, and p38, were sourced from Santa Cruz Biotechnology (Santa Cruz, CA, USA), and secondary antibodies were sourced from Jackson ImmunoResearch Laboratories (West Grove, PA, USA). Protein bands were visualised using enhanced chemiluminescence and imaged with a chemiluminescence imager [24].

### 2.10 Immunofluorescence

Immunofluorescence staining was performed to visualize the intracellular localization of NFκB p65 and cleaved GSDMD in A2E‐laden ARPE‐19 cells. After cell treatment, the cells were fixed in 10% neutral buffered formalin, permeabilized with 0.1% Triton X‐100, and blocked with 5% bovine serum albumin (BSA) at room temperature. Samples were then incubated overnight at 4°C with primary antibodies against NFκB, p‐NFκB, GSDMDC1, and GSDMD‐N (ABclonal, Woburn, USA). After washing with PBS, cells were incubated with Cy3‐conjugated secondary antibody (Affinipure Goat Anti‐Mouse or Anti‐Rabbit IgG; Jackson ImmunoResearch Laboratories, West Grove, PA, USA) in the dark. Nuclei were counterstained with 4′,6‐diamidino‐2‐phenylindole (DAPI, 1 μg/mL; AAT Bioquest, Sunnyvale, CA, USA). Fluorescence images were acquired on a Lionheart automated microscope (BioTek Instruments, Winooski, VT, USA). All immunofluorescence experiments were performed in at least three independent biological replicates per group, and fluorescence intensity was quantified using BioTek Gen5 Software (BioTek Instruments, Winooski, VT, USA).

### 2.11 Statistical Analysis

All data are presented as means ± standard deviations from at least three independent experiments. Differences between groups were evaluated using one‐way analyses of variance. *P*‐values of less than 0.05 were considered statistically significant.

## 3 Results

### 3.1 Irigenin Attenuates BL‐Induced Cytotoxicity in A2E‐laden ARPE‐19 Cells

**Figure 1:**
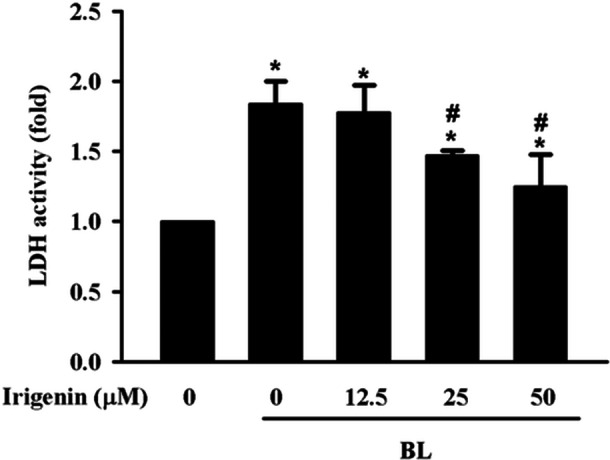
Irigenin reduces BL‐induced cytotoxicity in A2E‐laden ARPE‐19 cells as measured by lactose dehydrogenase release. Cells were pretreated with 0, 12.5, 25, or 50 µM irigenin before exposure to BL. Cytotoxicity was assessed following release of lactose dehydrogenase into the culture medium. Data are presented as means ± standard deviations from three independent experiments. ^⁎^ *p* < 0.05 indicates a significant difference compared with the control group, and ^#^ *p* < 0.05 indicates a significant difference compared with the BL treatment group.

LDH release, a marker of cellular damage, was measured to evaluate the protective effect of irigenin against BL‐induced damage in A2E‐laden ARPE‐19 cells. Exposure to BL for 15 min substantially increased LDH release compared with controls, indicating cytotoxicity. Pretreatment with irigenin for 1 h reduced LDH release in a dose‐dependent manner. At concentrations of 25 and 50 µM, LDH levels were significantly reduced, demonstrating that irigenin effectively mitigated BL‐induced cytotoxicity (Figure 1).

### 3.2 Irigenin Prevents BL‐Induced Cell Barrier Dysfunction in A2E‐laden ARPE‐19 Cells

**Figure 2:**
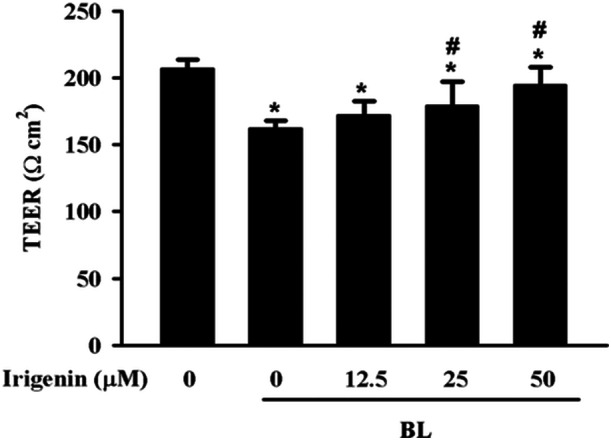
Irigenin reduces BL‐induced barrier dysfunction in A2E‐laden ARPE‐19 cells as measured by TEER. Cells were treated with irigenin at concentrations of 0, 12.5 µM, 25 µM, and 50 µM before exposure to BL. Data are presented as means ± standard deviations from three independent experiments. ^⁎^ *p* < 0.05 indicates a significant difference compared with the control group, and ^#^ *p* < 0.05 indicates a significant difference compared with the BL treatment group.

The protective effects of irigenin against in A2E‐laden ARPE‐19 cell barrier dysfunction under BL exposure were evaluated using TEER. Exposure to BL for 15 min significantly reduced TEER values, indicating compromised barrier function. Pretreatment with irigenin for 1 h preserved TEER values in a dose‐dependent manner. TEER levels were significantly maintained at 25 and 50 µM concentrations, demonstrating that irigenin effectively protected barrier integrity against BL‐induced damage (Figure 2).

### 3.3 Irigenin Reduces Bl‐Induced Production of Proinflammatory Cytokines in A2E‐laden ARPE‐19 Cells

**Figure 3:**
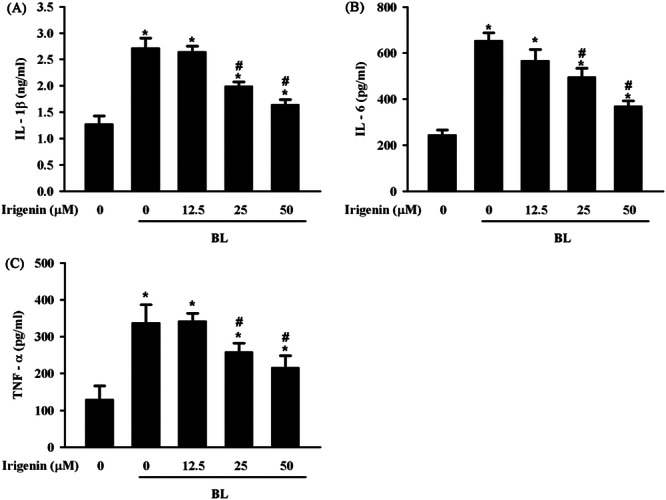
Irigenin reduces BL‐induced upregulation of pro‐inflammatory cytokines in A2E‐laden ARPE‐19 cells. Cells were pre‐treated with irigenin at concentrations of 12.5 µM, 25 µM, and 50 µM before exposure to BL, and IL‐1β (A), IL‐6 (B), and TNF‐α (C) levels in the culture supernatant were quantified using ELISA. Data are presented as means ± standard deviations from three independent experiments. ^⁎^ *p* < 0.05 indicates a significant difference compared with the control group, and ^#^ *p* < 0.05 indicates a significant difference compared with the BL treatment group.

The anti‐inflammatory effects of irigenin on BL‐induced cytokine production were assessed by measuring IL‐1β, IL‐6, and TNF‐α levels in the A2E‐laden ARPE‐19 cells. BL exposure substantially increased IL‐1β, IL‐6, and TNF‐α secretion compared with controls, indicating a strong inflammatory response. Pretreatment with 25 and 50 μM of irigenin considerably reduced cytokine production in a dose‐dependent manner, demonstrating the protective effect of irigenin in inhibiting proinflammatory cytokine release in A2E‐laden ARPE‐19 cells exposed to BL (Figure 3).

### 3.4 Irigenin Reduces BL‐Induced Expression of Nitric Oxide Synthase and Cyclooxygenase, and Inhibits NO Production in A2E‐laden ARPE‐19 Cells

**Figure 4:**
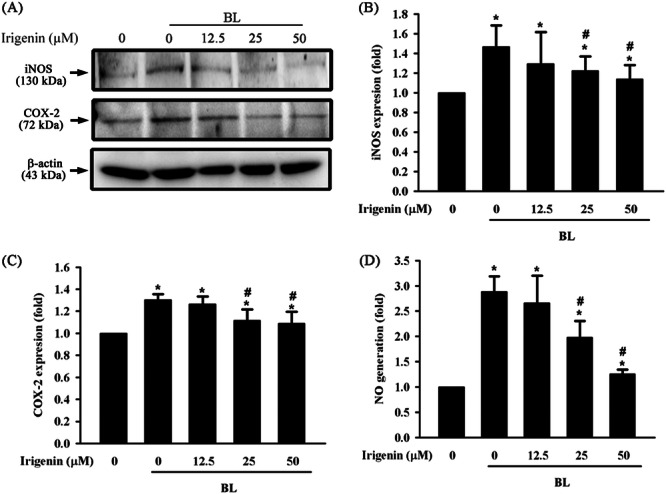
Irigenin inhibits BL‐induced upregulation of iNOS, COX‐2, and NO production in A2E‐laden ARPE‐19 cells. Cells were pre‐treated with irigenin at concentrations of 0, 12.5 µM, 25 µM, and 50 µM before exposure to BL. (A) iNOS and COX‐2 expression levels were assessed by Western blot. (B, C) Quantitative analysis of iNOS and COX‐2 protein expression levels. (D) NO production was quantified using the Griess assay. Data are presented as means ± standard deviations from three independent experiments. ^⁎^ *p* < 0.05 indicates a significant difference compared with the control group, and ^#^ *p* < 0.05 indicates a significant difference compared with the BL treatment group.

The anti‐inflammatory effects of irigenin were assessed by measuring nitric oxide synthase (iNOS), cyclooxygenase (COX‐2) expression, and NO production levels in A2E‐laden ARPE‐19 cells exposed to BL. BL exposure for 15 min significantly increased iNOS, COX‐2 expression, and NO production compared with controls. Pretreatment with irigenin for 1 h substantially reduced iNOS, COX‐2, and nitric oxide levels in a dose‐dependent manner. At 25 and 50 μM, these inflammatory markers were reduced, indicating that irigenin mitigated BL‐induced inflammatory responses (Figure 4).

### 3.5 Irigenin Suppresses BL‐Induced NLRP3 Inflammasome Activation in A2E‐laden ARPE‐19 Cells

**Figure 5:**
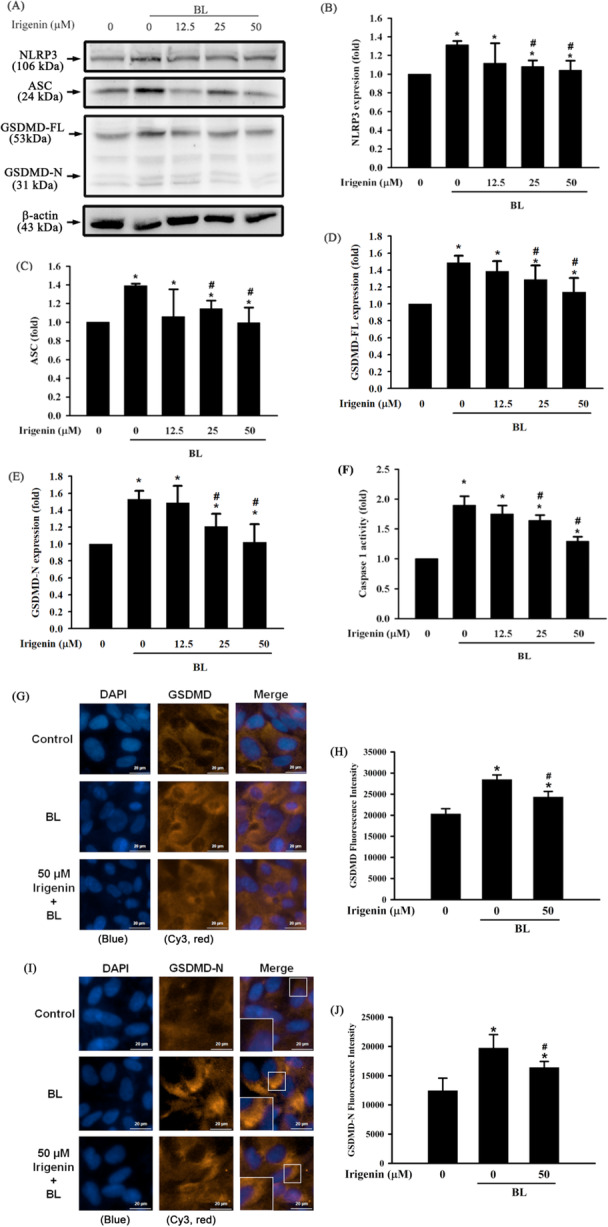
Irigenin attenuates NLRP3 inflammasome activation in A2E‐laden ARPE‐19 cells exposed to BL. Cells were pre‐treated with irigenin at concentrations of 0, 12.5 µM, 25 µM, and 50 µM before exposure to BL. (A) NLRP3, ASC, GSDMD‐FL, and GSDMD‐N expression levels were assessed by Western blot. (B–E) Quantitative analysis of NLRP3, ASC, GSDMD‐FL, and GSDMD‐N protein expression levels. (F) Caspase‐1 activity was measured using a colourimetric assay. Bar graphs indicate levels of caspase‐1 activity. (G) Immunofluorescence staining of GSDMDC1 (red, Cy3) and nuclei (blue, DAPI) in cells pretreated with 0 or 50 μM irigenin prior to BL exposure. (I) Immunofluorescence staining of GSDMD‐N (red, Cy3) and nuclei (blue, DAPI) in cells pretreated with 0 or 50 μM irigenin prior to BL exposure. High‐magnification images were included to visualize membrane localization of GSDMD‐N. Scale bar = 20 μm. (H, J) Quantitative analysis of Cy3 fluorescence intensity was performed. Data are presented as means ± standard deviations from three independent experiments. ^⁎^ *p* < 0.05 indicates a significant difference compared with the control group, and ^#^ *p* < 0.05 indicates a significant difference compared with the BL treatment group.

The protective effects of irigenin on BL‐induced pyroptosis were evaluated by analysing key markers of inflammasome activation. In the present study, the GSDMDC1 antibody targets a specific internal sequence of GSDMD located between amino acids 169 and 188, which falls within the N‐terminal domain. Since this epitope remains present in both the Gasdermin D full length (GSDMD‐FL) and Gasdermin D N‐terminal fragment (GSDMD‐N), the antibody is capable of recognizing both protein forms. Specifically, BL exposure for 15 min substantially increased the expression of NLRP3, apoptosis‐associated speck‐like protein containing a caspase activation and recruitment domain (ASC), GSDMD‐FL, and GSDMD‐N in addition to caspase‐1 activity, indicating NLRP3 inflammasome activation and pyroptotic cell death. Pretreatment with irigenin for 1 h substantially inhibited the upregulation of these markers in a dose‐dependent manner, indicating that irigenin suppressed the BL‐induced NLRP3 inflammasome activation in A2E‐laden ARPE‐19 cells (Figure 5A–F). Corroborating the above data, immunofluorescence analysis revealed that BL exposure markedly increased GSDMD and GSDMD‐N expression in both the cytoplasm and along the plasma membrane, indicating inflammasome activation and gasdermin‐mediated pore formation. Quantitative analysis of Cy3 fluorescence intensity confirmed a significant increase in GSDMD and GSDMD‐N staining following BL exposure compared with the control group (*p* < 0.05). In contrast, irigenin treatment (50 μM) markedly reduced both total cellular and membrane‐associated GSDMD‐N fluorescence intensity, indicating suppression of gasdermin‐mediated pore formation and pyroptotic signaling (Figure 5G–J).

### 3.6 Irigenin Attenuates BL‐Induced Activation of the NFκB Pathway in A2E‐laden ARPE‐19 Cells

**Figure 6:**
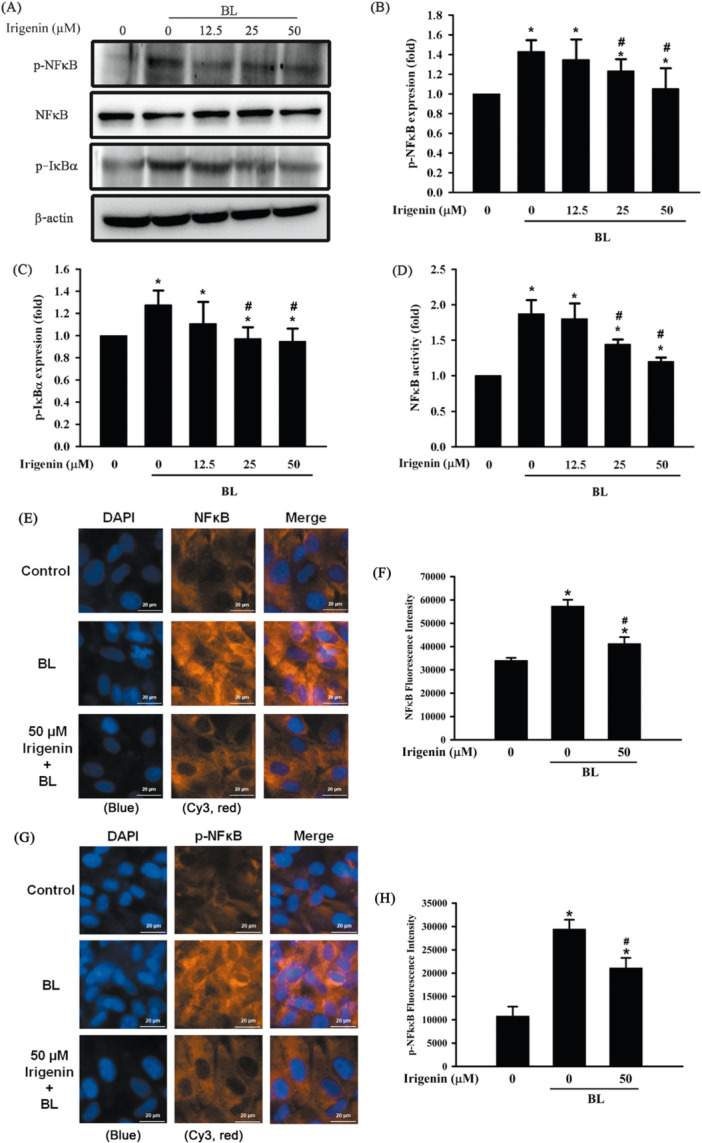
Irigenin attenuates NFκB pathway activation in A2E‐laden ARPE‐19 cells exposed to BL. Cells were pre‐treated with irigenin at concentrations of 12.5 µM, 25 µM, and 50 µM before exposure to BL. (A) Phosphorylation levels of NFκB and IκBα were assessed by Western blot. (B, C) Quantitative analysis of NFκB and IκBα phosphorylation levels. (D) NFκB activity was measured using a colorimetric assay. Bar graphs represent the quantification of NFκB activity. (E) Cells were pretreated with 0 and 50 μM irigenin before BL exposure. After fixation, cells were stained for NFκB (red, Cy3) and nuclei (blue, DAPI), and imaged using a Lionheart automated microscope. (G) Cells were pretreated with 0 and 50 μM irigenin before BL exposure. After fixation, cells were stained for p‐NFκB (red, Cy3) and nuclei (blue, DAPI). Scale bar = 20 μm. (F, H) Quantitative analysis of Cy3 fluorescence intensity was performed. Data are presented as means ± standard deviations from three independent experiments. ^⁎^ *p* < 0.05 indicates a significant difference compared with the control group, and ^#^ *p* < 0.05 indicates a significant difference compared with the BL treatment group.

The effect of irigenin on BL‐induced activation of the NFκB pathway was assessed by separately analyzing IκB‐α and NFκB phosphorylation as well as NFκB nuclear translocation in A2E‐laden ARPE‐19 cells. Exposure to BL for 15 min significantly increased IκB‐α and NFκB phosphorylation, reflecting pronounced pathway activation. Concurrently, BL exposure markedly enhanced NFκB nuclear translocation. Pretreatment with irigenin for 1 h led to a significant, dose‐dependent reduction in both IκB‐α and NFκB phosphorylation, with significant decreases observed at 25 and 50 μM (Figure 6A–C). Similarly, irigenin pretreatment substantially reduced NFκB nuclear translocation, with significant effects at 25 and 50 μM, indicating that irigenin effectively suppressed BL‐induced NFκB pathway activation (Figure 6D). Corroborating the above data, immunofluorescence analysis showed that BL exposure triggered a prominent nuclear translocation of NFκB, whereas treatment with irigenin at 50 μM markedly diminished this nuclear accumulation in A2E‐laden ARPE‐19 cells (Figure 6E). In parallel, immunofluorescence analysis using p‐NFκB–specific antibodies demonstrated a prominent increase in nuclear p‐NFκB immunoreactivity following BL exposure, as evidenced by intensified Cy3 red fluorescence within the nucleus (Figure 6G). Quantitative analysis of Cy3 fluorescence intensity confirmed a significant elevation of nuclear NFκB and p‐NFκB levels compared with the control group (*p* < 0.05). In contrast, pretreatment with irigenin (50 μM) markedly attenuated BL‐induced NFκB and p‐NFκB nuclear accumulation and significantly reduced nuclear Cy3 fluorescence intensity, indicating effective suppression of NFκB activation and transcriptional signaling (Figure 6F,H).

### 3.7 Irigenin Attenuates BL‐Induced Phosphorylation of p38 MAPK in A2E‐laden ARPE‐19 Cells

**Figure 7:**
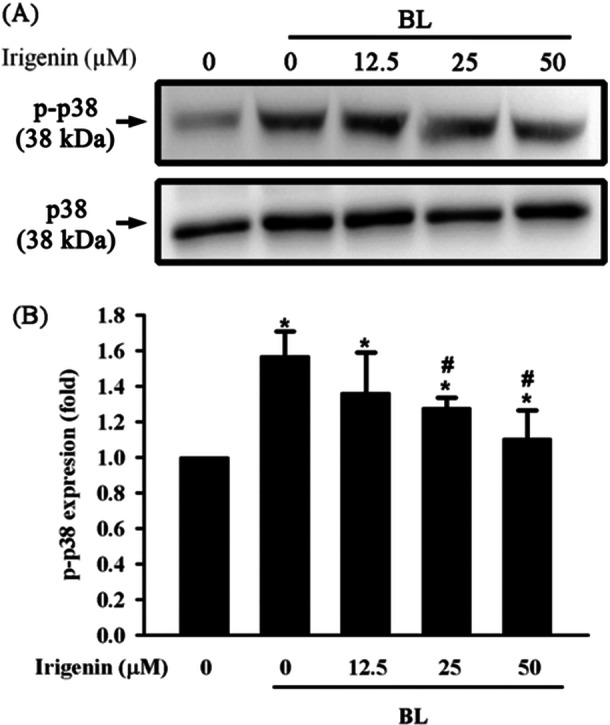
Irigenin attenuates p38 MAPK phosphorylation in A2E‐laden ARPE‐19 cells exposed to BL. Cells were pre‐treated with irigenin at concentrations of 12.5 µM, 25 µM, and 50 µM before exposure to BL. (A) Phosphorylation levels of p38 MAPK were assessed by Western blot. (B) Quantitative analysis of p38 MAPK phosphorylation levels. Data are presented as means ± standard deviations from three independent experiments. ^⁎^ *p* < 0.05 indicates a significant difference compared with the control group, and ^#^ *p* < 0.05 indicates a significant difference compared with the BL treatment group.

The inhibitory effect of irigenin on BL‐induced p38 phosphorylation in A2E‐laden ARPE‐19 cells was evaluated using Western blot analysis. Exposure to BL for 15 min significantly increased p38 phosphorylation compared with the control group, indicating the activation of the p38 MAPK pathway. Pretreatment with irigenin for 1 h led to a significant, dose‐dependent reduction in p38 phosphorylation. Notably, at concentrations of 25 and 50 μM, the levels of p38 phosphorylation were significantly lower than those in the BL‐exposed group, demonstrating that irigenin effectively suppressed BL‐induced p38 pathway activation (Figure 7).

## 4 Discussion

AMD is a complex retinal disorder influenced by environmental and molecular factors, with inflammation as a central mechanism [25]. High‐energy BL damages retinal cells, increasing stress and impairing RPE function [26]. A2E, a phototoxic byproduct of the visual cycle that accumulates in RPE cells, is a major lipofuscin constituent and a key contributor to AMD pathogenesis [27, 28]. While this study primarily focused on the A2E‐laden condition to model dry AMD, we also conducted preliminary experiments using BL exposure alone, without A2E pretreatment. Under the same irradiation parameters (6,000 lux for 15 min), BL alone did not cause significant changes in cell damage and oxidative stress (Supporting Information: Figure S1). This can be attributed to the combined effects of the low intensity and the brief duration of BL exposure. These findings further support that A2E plays a critical role in sensitising RPE cells to BL‐induced damage. Specifically, exposure to BL causes A2E photooxidation, producing reactive intermediates that induce RPE cell death, cause inflammation, and compromise RPE barrier integrity [29]. Irigenin, with its anticancer, antioxidant, and anti‐inflammatory properties, exerts protective effects in several disease models, such as those of doxorubicin‐induced cardiotoxicity, MPP^+^‐induced neurotoxicity through the activation of the Keap1/Nrf2 pathway in BV‐2 cells, and angiotensin II‐induced oxidative damage through the activation of the Nrf2 pathway in human umbilical vein endothelial cells [30, 31]. This study utilized in A2E‐laden ARPE‐19 cells as an in vitro model to simulate AMD conditions. The results of an LDH assay revealed substantially increased cytotoxicity and decreased TEER, indicating compromised RPE barrier integrity. Pretreatment with irigenin reduced this cytotoxicity and preserved TEER in a dose‐dependent manner, demonstrating irigenin's potential as a preventive agent against BL‐induced damage in AMD. Therefore, this study is among the first to demonstrate irigenin's protective effects against AMD and explore its molecular mechanisms.

Uncontrolled inflammation is a key pathological feature of AMD that substantially contributes to disease progression [32]. In aging eyes, RPE cells dysregulate pro‐ and anti‐inflammatory cytokines, leading to persistent inflammation [33]. Other studies have reported BL‐induced inflammation in A2E‐laden ARPE‐19 cells [34]. Irigenin may mitigate diffuse alveolar injury and pulmonary oedema in acute lung injury through its anti‐inflammatory properties [35]. Irigenin may also inhibit angiotensin‐II‐induced inflammation in human umbilical vein endothelial cells [31]. In the present study, pretreatment with irigenin considerably reduced BL‐induced IL‐1β, IL‐6, and TNF‐α production in A2E‐laden ARPE‐19 cells, highlighting its potential to modulate AMD‐related inflammation.

NO generation is closely associated with iNOS and COX‐2 expression and plays a crucial role in inflammatory pathways [36]. Specifically, upregulated iNOS increases NO production and modulates immune responses but can cause cytotoxicity when excessive; upregulated iNOS also increases COX‐2 expression, contributing to proinflammatory mediator synthesis and amplifying inflammation [37]. This study provides the first evidence of the protective effects of irigenin against BL‐induced inflammation in A2E‐laden ARPE‐19 cells through the inhibition of nitric oxide, iNOS, and COX‐2, indicating that treatment with irigenin mitigates BL‐induced inflammatory responses.

Pyroptosis, a form of programmed cell death triggered by inflammatory signals, is marked by inflammasome activation, GSDMD‐FL cleavage, and the release of proinflammatory cytokines [38]. At the molecular level, pyroptotic execution is mediated by caspase‐1–dependent cleavage of GSDMD, generating the GSDMD‐N that inserts into the plasma membrane. Other studies have revealed that BL exposure in A2E‐laden RPE cells can induce pyroptosis [3]. The western blot assay data presented in this study corroborated the induction of pyroptosis by demonstrating a significant upregulation of NLRP3, ASC, GSDMD‐FL, and the activated N‐terminal fragment GSDMD‐N in A2E‐laden ARPE‐19 cells subjected to BL exposure. Furthermore, findings from the immunofluorescence assay reinforced these observations, revealing that the upregulation of GSDMD‐N is predominantly localized within the cytoplasm and along the plasma membrane. Quantitative analysis of Cy3 fluorescence intensity further confirmed a significant increase in membrane‐associated GSDMD‐N following BL exposure. In contrast, treatment with irigenin resulted in a marked reduction of these signals, including both total cellular and membrane‐localized GSDMD‐N, thereby substantiating the biochemical findings. Collectively, these results indicate that irigenin effectively inhibits inflammasome activation, gasdermin‐mediated membrane pore formation, pyroptotic cell death, and the associated inflammatory response in A2E‐laden ARPE‐19 cells following exposure to blue light. This investigation primarily focused on the NLRP3 inflammasome; however, emerging evidence suggests that other inflammasome complexes, such as AIM2, may significantly contribute to retinal inflammation and degeneration. AIM2 can recognize cytosolic double‐stranded DNA and has been implicated in sterile inflammatory responses across various models of retinal injury, particularly those characterized by photoreceptor stress and oxidative DNA damage. Although the current study did not directly assess the activation of AIM2, the cleavage of caspase‐1 observed serves as a functional indicator of inflammasome activation. Previous studies have established that caspase‐1 cleavage serves as a reliable marker of inflammasome activation and the subsequent initiation of pyroptotic signaling, particularly when accompanied by GSDMD‐N activation and IL‐1β release [39, 40, 41, 42]. Thus, the detection of caspase‐1 and GSDMD‐N in our experimental model indicates that irigenin effectively inhibits inflammasome‐mediated pyroptosis triggered under BL and A2E conditions. Taken together, these findings identify GSDMD‐N–dependent pyroptosis as a key pathological event in BL‐exposed A2E‐laden ARPE‐19 cells and demonstrate, for the first time, that irigenin suppresses this process. Thus, this is the first study to demonstrate irigenin's inhibition of BL‐induced pyroptosis, indicating its potential to protect against A2E‐laden ARPE‐19 cell damage.

The NFκB signalling pathway, a critical mediator of inflammation, is crucial to pyroptosis and other inflammatory responses in various pathological conditions, including AMD [43]. The NFκB family consists of five proteins—p50, p52, p65 (RelA), RelB, and c‐Rel—with p65 being the most well‐studied for its central role in regulating proinflammatory gene expression [44]. When this pathway is exposed to inflammatory stimuli, p65 is released from the NFκB‐IκB complex into the cytosol, undergoes phosphorylation at specific serine residues, and is subsequently translocated to the nucleus, where it activates proinflammatory genes that encode proinflammatory mediators such as cytokines and adhesion molecules [45]. Thus, phosphorylation‐dependent activation and nuclear translocation of NFκB p65 represent hallmarks of functional NFκB signalling. Several studies have demonstrated that BL‐induced inflammation in A2E‐laden ARPE‐19 cells is mediated by NFκB signalling [46]. The current study demonstrated that irigenin pretreatment significantly reduces the BL‐induced inflammatory response by suppressing NFκB pathway activation, as evidenced by attenuated phosphorylation of both IκBα and NFκB, as well as reduced nuclear accumulation of phosphorylated NFκB, thereby confirming irigenin's protective effects against BL‐induced inflammation in A2E‐laden ARPE‐19 cells at both the phosphorylation and nuclear translocation levels.

Extracellular and intracellular stimuli activate the MAPK signalling pathway and regulate key cellular processes such as proliferation, differentiation, survival, and apoptosis [47]. The role of this pathway in inflammation also links it to various inflammatory diseases [48]. In BL‐induced RPE cell models, MAPK is an upstream mediator of NFκB activation, indicating its critical role in inflammatory responses [49]. Previous studies have demonstrated that irigenin substantially inhibits the MAPK pathway in lipopolysaccharide‐induced mouse models [35]. Additionally, the present study demonstrated that pretreatment with irigenin considerably reduced BL‐induced p38 MAPK phosphorylation in A2E‐laden ARPE‐19 cells.

While the present study has several advantages, it is still important to recognize several limitations. The in vitro A2E‐laden ARPE‐19 model is the feasible simulation of the oxidative stress and inflammatory conditions characteristic of dry AMD. Nonetheless, it does not adequately encompass the intricate complexities of the retinal microenvironment [12, 13, 14, 15, 16, 17]. In particular, it does not capture the cellular and vascular interactions among the RPE, Bruch's membrane, and choroidal vasculature, nor the angiogenic processes that drive the progression to wet AMD [15, 16, 17]. Geographic atrophy may advance to neovascular AMD through VEGF‐dependent or ‐independent mechanisms, with VEGF serving as a critical mediator of this transition [50, 51, 52]. Given irigenin's potent suppression of inflammation and pyroptosis, it is plausible that it may also modulate VEGF signaling. However, this possibility has not been addressed in the current work. Future studies incorporating co‐culture systems validation are therefore warranted to extend these findings to angiogenesis and to consolidate mechanistic insights.

Furthermore, in vitro systems cannot fully recapitulate the immunological and structural complexity of the retina, where innate immunity and cell–cell interactions critically modulate disease progression. To address this problem, our earlier in vivo study using a mouse model of retinal degeneration caused by BL found that irigenin reduces structural damage to the retina. It also lowers oxidative stress and enhances antioxidant defenses by stimulating HO‐1 and NQO‐1, which emphasizes its importance for physiological health [14]. Beyond pyroptosis, there is increasingly recognized that other regulated forms of cell death, such as apoptosis, necroptosis, and ferroptosis, are also elicited in RPE cells under BL‐induced damage [53, 54, 55]. Nonetheless, additional in vivo studies are necessary to examine inflammasome activation pathways, including NLRP3, caspase‐1, and GSDMD cleavage, and to explore other regulated cell death programs, such as apoptosis, necroptosis, and ferroptosis, that may act in concert with pyroptosis. Such efforts will be critical to fully delineate the cytoprotective mechanisms of irigenin and to establish its translational potential for both dry and wet AMD.

## 5 Conclusion

**Figure 8:**
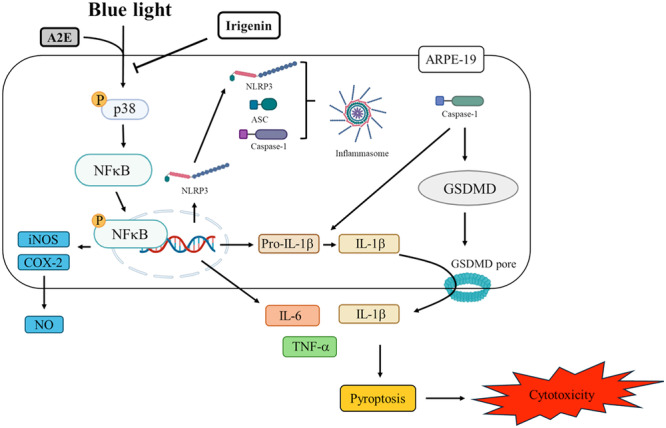
Schemes of irigenin alleviate BL‐induced pyroptosis by upregulating p38 MAPK and NFκB pathways.

The present study further demonstrated that irigenin mitigates BL‐induced damage in A2E‐laden ARPE‐19 cells by modulating inflammatory and pyroptotic pathways. Specifically, irigenin inhibits p38 MAPK phosphorylation and downregulates NFκB activation, reducing proinflammatory cytokine release and preventing pyroptosis (Figure 8). These findings suggest irigenin's potential to treat AMD by mitigating pyroptosis and inflammation.

## Author Contributions

**I‐Li Su:** conceptualization, data curation, funding acquisition, investigation, project administration, supervision writing ‐ review & editing. **Kun‐Lin Yeh:** conceptualization, data curation, investigation, methodology, software, visualization, writing ‐ original draft. **Chien‐Ying Lee:** data curation, methodology, software. **Chen‐Yu Chiang:** investigation, methodology, software, validation. **Chun‐Jung Chen:** conceptualization, visualization, writing ‐ original draft. **Wen‐Ying Chen:** conceptualization, visualization, writing ‐ original draft. **Ching‐Chi Tseng:** conceptualization, methodology, visualization, writing ‐ original draft. **Yin‐Che Lu:** conceptualization, funding acquisition, project administration, supervision, writing ‐ review & editing. **Yu‐Hsiang Kuan:** conceptualization, funding acquisition, project administration, supervision, writing ‐ review & editing.

## Conflicts of Interest

The authors declare no conflicts of interest.

## Supporting information


**Supplemental Figure S1:** Effects of BL exposure on ARPE‐19 cells with or without A2E pretreatment.

## Data Availability

Data available on request from the authors. The data that support the findings of this study are available from the corresponding author upon reasonable request.
