# Supplementary material for: Irigenin Modulates BL‐Induced Pyroptosis in Retinal Pigment Epithelial Cells Through p38 MAPK and NFκB Pathways
Source: J Biochem Mol Toxicol. 2026 Feb 5;40(2):e70723. doi: 10.1002/jbt.70723 (PMC12875303; doi:10.1002/jbt.70723)

**Supplemental Figure S1 Effects of BL exposure on ARPE-19 cells with or without A2E pretreatment.**

ARPE-19 cells were pretreated with or without 20 μM A2E for 24 h, followed by exposure to BL irradiation (430 nm, 6000 lux) for 15 min. (A) Cytotoxicity was assessed following release of lactose dehydrogenase into the culture medium. (B) Intracellular ROS levels were measured using the 2'-7'-Dichlorodihydrofluorescein diacetate (DCFH-DA) reagents. Data are presented as mean ± SD (n = 3). ^⁎^ *P* < .05 indicates a significant difference compared with the control group. In cells without A2E, BL exposure did not significantly alter cell viability or ROS levels compared to the non-irradiated control. In contrast, A2E-pretreated cells exhibited a significant decrease in cell viability and a marked increase in ROS production upon BL exposure. These findings confirm that A2E loading is essential for mediating BL-induced cytotoxicity and oxidative stress under the present experimental conditions.


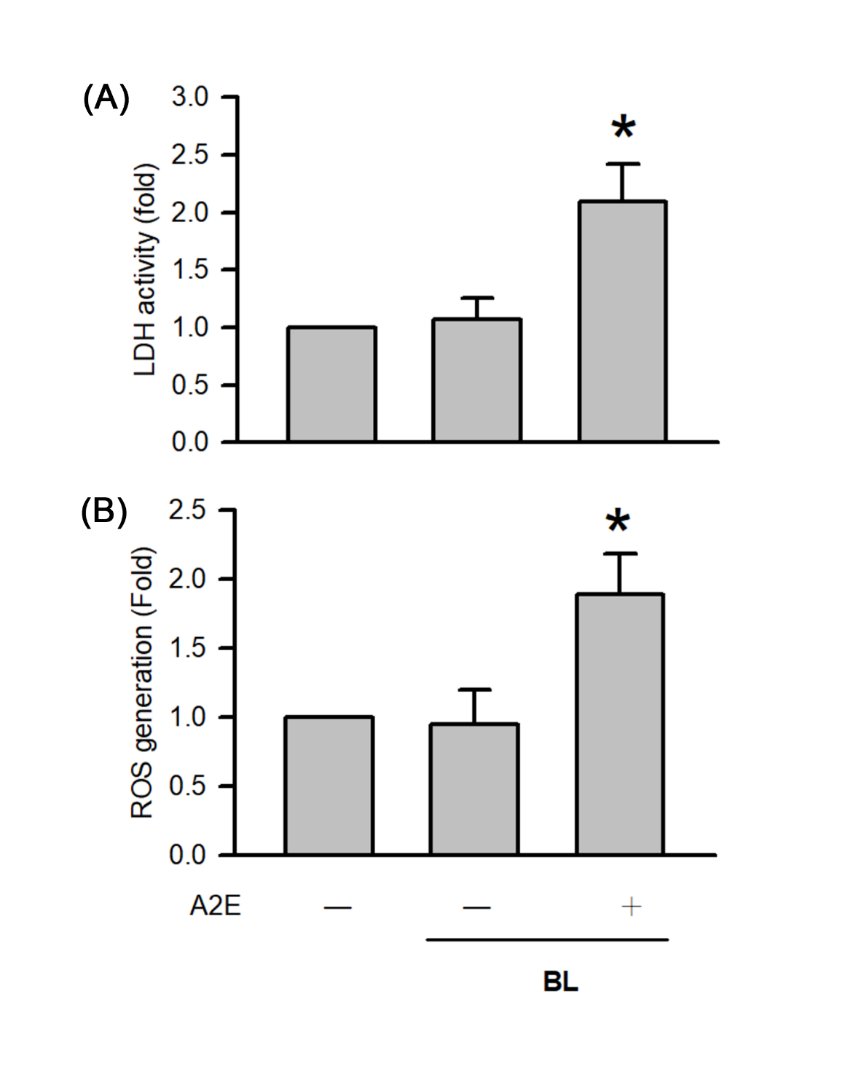

Supplement: Supplementary file 1 — Supplemental Figure S1: Effects of BL exposure on ARPE‐19 cells with or without A2E pretreatment. [file JBT-40-e70723-s001.docx]
